# Supplementary material for: C-C motif chemokine receptor 2 and 7 synergistically control inflammatory monocyte recruitment but the infecting virus dictates monocyte function in the brain
Source: Commun Biol. 2024 Apr 24;7:494. doi: 10.1038/s42003-024-06178-6 (PMC11043336; doi:10.1038/s42003-024-06178-6)
Supplement: Supplementary file 2 — Supplementary Information [file 42003_2024_6178_MOESM2_ESM.pdf]

Supplementary information

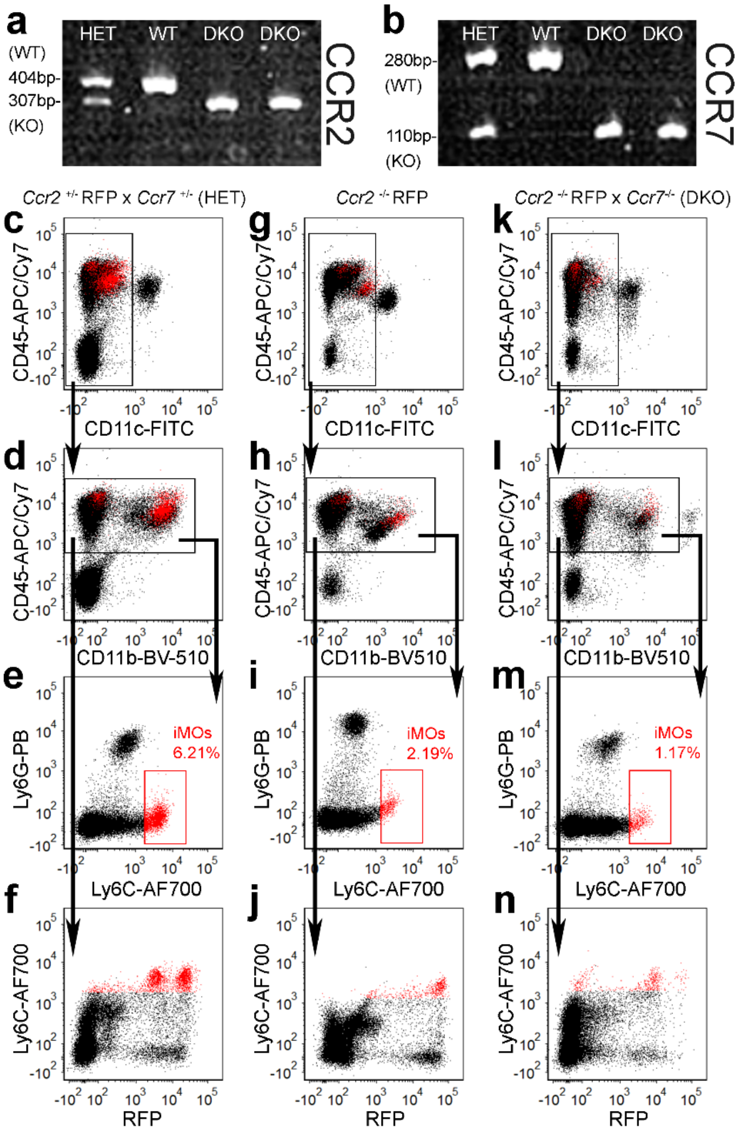

<u>Supplementary Figure 1. Generation of *Ccr2*$^{-/-}$ RFP *Ccr7*$^{-/-}$ double knockout (DKO) mice and gating strategy for iMOs.</u> PCR analysis of (**a**) *Ccr2* and (**b**) *Ccr7* from tail biopsies performed on HET, WT and DKO mice. The gel was run top to bottom as indicated by the product sizes. The wildtype (WT) allele is indicated by the upper band for both *Ccr2* and *Ccr7* and are present only in HET and WT samples. The knockout (KO) allele is indicated by the lower band and is present only in the HET and DKO. Representative flow cytometric cell phenotyping plots with gating strategy for iMOs from blood samples from mock inoculated

(**c**-**f**) HET, (**g**-**j**) *Ccr2*$^{-/-}$ RFP and (**k**-**n**) DKO mice are shown. (**c**, **g** and **k**) CD11c+ dendritic cells were excluded from analysis and (**c, g** and **k**) CD45+ populations were analyzed for (**e**, **i** and **m**) Ly6c^{hi} and (**f**, **j** and **n**) CCR2^{+} positive iMOs. Red labeled events indicate iMOs in all plots. Notice that the populations of (**c** v. **k**) CD11c^{+} dendritic cells and (**e** v. **m**) Ly6G^{+} neutrophils are similar in HET v. DKO mice.

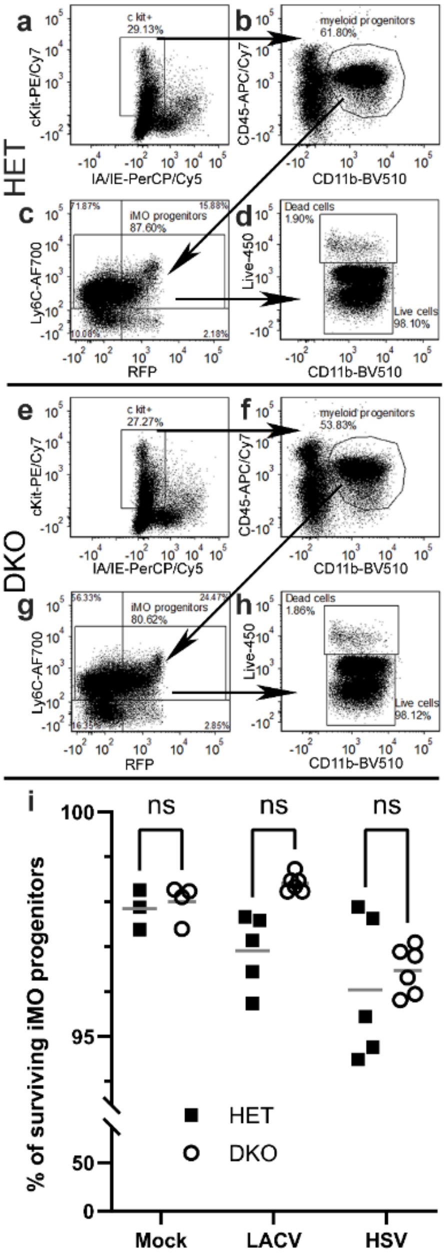

Supplementary Figure 2. iMO progenitor populations in the bone marrow are not altered by *Ccr2*$^{-/-}$ RFP *Ccr7*$^{-/-}$ double knockout. Representative flow cytometric cell phenotyping plots of iMOs from bone marrow samples from mock inoculated (**a-d**) HET and (**e-h**) DKO mice. (**a** and **e**) ckit+ bone marrow progenitors were gated on and (**b** and **f**) CD45+ CD11b+ myeloid progenitors and specifically (**c** and **g**) iMO progenitors were identified. (**d** and **i**) Vital dye was used to identify live and dead cells from each sample and (**i**) the percent surviving iMO progenitors were quantified from mock, HSV and LACV infected mice. A two-way ANOVA with an alpha of 0.05 with a Sidak's multiple comparisons test was performed to examine differences in iMO survival between HET and DKO mice with the following statistics:HSV:DKO vs. HET, t=0.8659, df=23, p=0.995; LACV:DKO vs. HET, t=3.014, df=23, p=0.0888 and Mock:DKO vs. HET, t=0.2571, df=23, p=>0.9999. Results from individual animals are plotted with the horizontal bars representing the mean for each group.

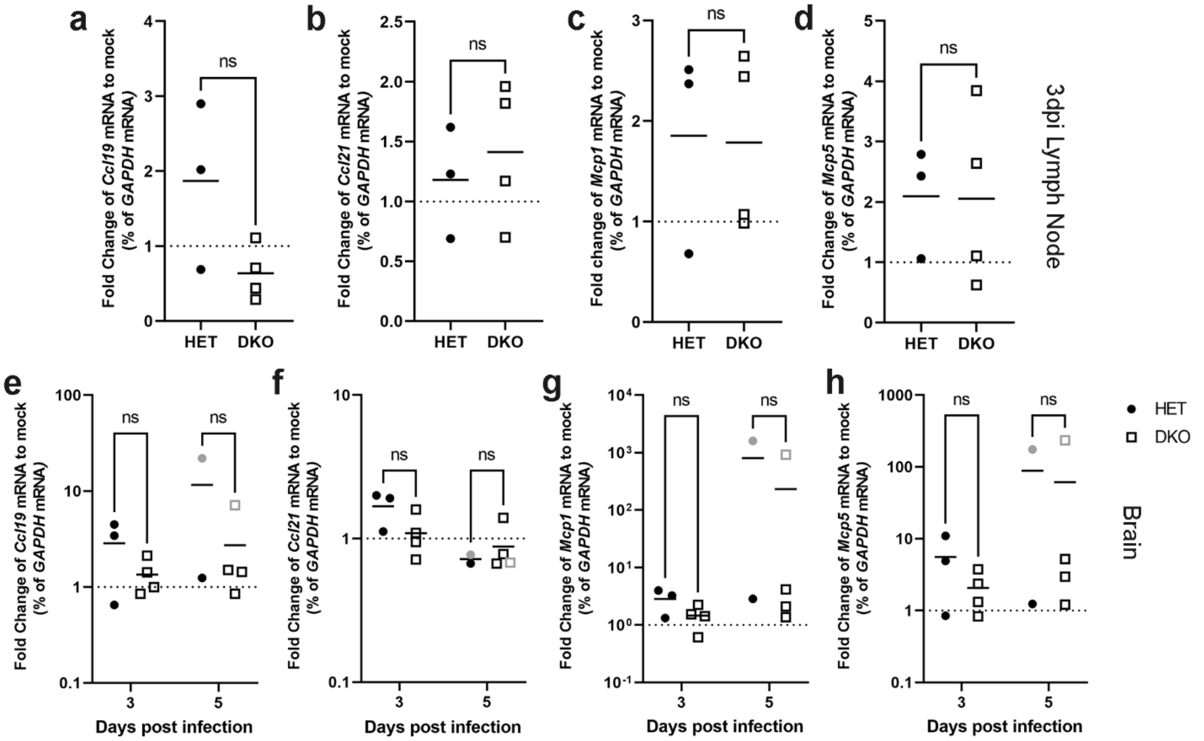

Supplementary Figure 3. Transcripts of CCR2 and CCR7 ligands are expressed at similar levels in lymph node and brain in HET and DKO mice during LACV infection. mRNA expression of the CCR7 ligands (**a** and **e**) *Ccl19* and (**b** and **f**) *Ccl21* and the CCR2 ligands (**c** and **g**) *Mcp1* and (**d** and **h**) *Mcp5* were evaluated in the (**a-d**) 3dpi lymph node or (**e-h**) 3 and 5dpi brain of HET and DKO mice infected IP with 10^3 LACV. For all graphs, fold change relative to mock is plotted on the y-axis Each individual data point represents an individual mouse. Gray points in E-H indicate clinical animals. Dotted lines indicate a fold change of 1. Lymph node expression was compared using a two-tailed, unpaired t-test. Brain expression was compared using a 2-way ANOVA with a Tukey multiple comparisons test between time points. ns= not significant.

Supplementary Table 1.

Gene	Primer	Sequence 5'-3'
Ccr2	Common forward	CCAAAGATGGGGATACTGCT
	Wild type reverse	GATGGCCAAGTTGAGCAGAT
	Mutant reverse	TACTGGAACTGAGGGGACAG
Ccr7	Common forward	TAAGGGCATCTTTGGCATCT
	Wild type reverse	GGTGATCAAGGCCTCCACT
	Mutant reverse	AGACTGCCTTGGGAAAAGCG

Supplementary Table 1. Genotyping primers used for the generation of $Ccr2^{-/-}$ RFP x $Ccr7^{-/-}$ DKO mice.

Supplementary Table 2.

Target	Primer	Sequence 5'-3'
LACV	Forward	ATTCTACCCGCTGACCATTG
	Reverse	GTGAGAGTGCCATAGCGTTG
Ccl19	Forward	GACCTAACCCAGCCAAGTCT
	Reverse	TGCAGTCTAGCCACAGAGAG
Ccl21	Forward	CAAGGGCTGCAAGAGAACTG
	Reverse	GTGAACCACCCAGCTTGAAG
Mcp1	Forward	CCCACTCACCTGCTACT
	Reverse	TCTGGACCCATTCCTTCTTG
Mcp5	Forward	AGCTACCACCATCAGTCCTCA
	Reverse	CGGACGTGAATCTTCTGCTT

Supplementary Table 2. qRT primers used for analysis of virus and chemokine expression in tissues.
