# Supplementary material for: C-C motif chemokine receptor 2 and 7 synergistically control inflammatory monocyte recruitment but the infecting virus dictates monocyte function in the brain
Source: Commun Biol. 2024 Apr 24;7:494. doi: 10.1038/s42003-024-06178-6 (PMC11043336; doi:10.1038/s42003-024-06178-6)
Supplement: Supplementary file 3 — Description of Additional Supplementary Files [file 42003_2024_6178_MOESM3_ESM.pdf]

Description of Additional Supplementary Files

File name: Supplementary Data 1

Description: RT2 Profiler PCR Array of 370 GPCR transcripts from FACs isolated iMOs from LACV and HSV infected mouse brain.

File name: Supplementary Data 2

Description: Numerical source data for the Figures.
