# Supplementary material for: C-C motif chemokine receptor 2 and 7 synergistically control inflammatory monocyte recruitment but the infecting virus dictates monocyte function in the brain
Source: Commun Biol. 2024 Apr 24;7:494. doi: 10.1038/s42003-024-06178-6 (PMC11043336; doi:10.1038/s42003-024-06178-6)
Supplement: Supplementary file 6 — Reporting Summary [file 42003_2024_6178_MOESM6_ESM.pdf]

nature portfolio

Corresponding author(s): Clayton Winkler

Last updated by author(s): Apr 3, 2024

Reporting Summary

Nature Portfolio wishes to improve the reproducibility of the work that we publish. This form provides structure for consistency and transparency in reporting. For further information on Nature Portfolio policies, see our Editorial Policies and the Editorial Policy Checklist.

Statistics

For all statistical analyses, confirm that the following items are present in the figure legend, table legend, main text, or Methods section.

n/a	Confirmed	
☐	☒	The exact sample size (n) for each experimental group/condition, given as a discrete number and unit of measurement
☐	☒	A statement on whether measurements were taken from distinct samples or whether the same sample was measured repeatedly
☐	☒	The statistical test(s) used AND whether they are one- or two-sided<br>*Only common tests should be described solely by name; describe more complex techniques in the Methods section.*
☐	☒	A description of all covariates tested
☐	☒	A description of any assumptions or corrections, such as tests of normality and adjustment for multiple comparisons
☐	☒	A full description of the statistical parameters including central tendency (e.g. means) or other basic estimates (e.g. regression coefficient) AND variation (e.g. standard deviation) or associated estimates of uncertainty (e.g. confidence intervals)
☐	☒	For null hypothesis testing, the test statistic (e.g. F, t, r) with confidence intervals, effect sizes, degrees of freedom and P value noted<br>*Give P values as exact values whenever suitable.*
☒	☐	For Bayesian analysis, information on the choice of priors and Markov chain Monte Carlo settings
☒	☐	For hierarchical and complex designs, identification of the appropriate level for tests and full reporting of outcomes
☒	☐	Estimates of effect sizes (e.g. Cohen's d, Pearson's r), indicating how they were calculated

Our web collection on statistics for biologists contains articles on many of the points above.

Software and code

Policy information about availability of computer code

Data collection	Softwares and their version used for data collection:<br>1. BD FACSDiva Software v9<br>2. Viia7 Software v1.2.2<br>3. RSEM v1.3.3<br>4. DESeq2 v3.18
Data analysis	Softwares and their version used for data analysis:<br>1. FCS Express Research Edition v.5<br>2. Office Excel v16<br>3. GraphPad Prism v9.3.1<br>4. Clustvis written in R (R package v. 0.10.2.1)

For manuscripts utilizing custom algorithms or software that are central to the research but not yet described in published literature, software must be made available to editors and reviewers. We strongly encourage code deposition in a community repository (e.g. GitHub). See the Nature Portfolio guidelines for submitting code & software for further information.

Data

Policy information about availability of data

All manuscripts must include a data availability statement. This statement should provide the following information, where applicable:
- Accession codes, unique identifiers, or web links for publicly available datasets
- A description of any restrictions on data availability
- For clinical datasets or third party data, please ensure that the statement adheres to our policy

Source data for Figure 1e are provided in Supplementary Table 1. RNA-seq analysis data from Figures 5 and 6 have been upload to NCBI Geo and can be accessed with Accession # GSE254432. All other data that support the findings of this study are available from the corresponding author (CWW) upon reasonable request.

Human research participants

Policy information about studies involving human research participants and Sex and Gender in Research.

Reporting on sex and gender	NA
Population characteristics	NA
Recruitment	NA
Ethics oversight	NA

Note that full information on the approval of the study protocol must also be provided in the manuscript.

Field-specific reporting

Please select the one below that is the best fit for your research. If you are not sure, read the appropriate sections before making your selection.

☒ Life sciences ☐ Behavioural & social sciences ☐ Ecological, evolutionary & environmental sciences

For a reference copy of the document with all sections, see nature.com/documents/nr-reporting-summary-flat.pdf

Life sciences study design

All studies must disclose on these points even when the disclosure is negative.

Sample size	Final sample size was determined by multiple factors. For the RT2 Profiler PCR array and RNA-seq analysis, the number of mice used in each group (3) was dictated respectively by the array and the sequencing chip to reach an adequate depth sequencing coverage . For flow cytometry, phagocytosis, qRT and survival analyses, an initial power analysis was performed to estimate the number of animals required to detect a specified mean fold change and standard deviation, that were based on results from previous experiments, between groups assuming an alpha of 0.05 and 80% power. These estimates were 6 mice per group for cytometry, phagocytosis and qRT analyses and 14 mice for survival analyses. The final number of mice used in each experimental group was further dictated by the accumulated mean and variance within a group as additional replicates were added. If means and variance were consistent within a group after analyzing 5 or more mice, no additional mice were used. The breeding success of a specific strain, such as Ccr7-/- mice also limited the number of mice that were available for analysis.
Data exclusions	No data were excluded from the analysis.
Replication	With the exception of the RT2 Profiler PCR array and RNA-seq analysis, which was performed on a single group of mice infected at the same time, each experimental group is comprised of animals from multiple different litters, infected at different times. At a minimum groups contained animals from three separate infections and litters with the majority of experimental groups be comprised of animals from 4-5 separate infections and litters.
Randomization	Mice were randomized between mock or infected groups for each litter used. Animals of either sex were split as evenly as possible within each litter between the mock and infected groups.
Blinding	For the RT2 Profiler PCR array, RNA-seq, flow cytometry, phagocytosis and qRT experiments, the investigator was responsible for injection and harvesting of tissues and was thus unblinded for this process. However, after tissue collection, all experimental animal tissues were assigned a de-identified sequential tracking number which followed the specific tissue through the processing, experimentation and analysis pipeline. Only after the final analysis was completed were the tracking numbers cross-reference with the experimental groups to unblind the investigator.<br>For survival studies, the investigator was not blinded because they were responsible for randomizing groups by sex and treatment group and performed the injections. However, monitoring animals for development of disease is not subjective and any clinical signs of neurological disease including ataxia, seizures or paralysis results in a clinical score. Clinical disease progresses rapidly in mice infected with LACV and a

mouse showing mild neurological signs will progress to severe disease within a matter of hours, thus a clinical score is consistently accurate to that day.

Reporting for specific materials, systems and methods

We require information from authors about some types of materials, experimental systems and methods used in many studies. Here, indicate whether each material, system or method listed is relevant to your study. If you are not sure if a list item applies to your research, read the appropriate section before selecting a response.

Materials & experimental systems

n/a	Involved in the study	
☐	☒	Antibodies
☐	☒	Eukaryotic cell lines
☒	☐	Palaeontology and archaeology
☐	☒	Animals and other organisms
☒	☐	Clinical data
☒	☐	Dual use research of concern

Methods

n/a	Involved in the study	
☒	☐	ChIP-seq
☐	☒	Flow cytometry
☒	☐	MRI-based neuroimaging

Antibodies

Antibodies used	All antibodies used are displayed in Table 2 and are listed below with antigen, fluorochrome, clone, source, catalog# and lot#.<br>1) CD45, APC/Cy7, 30-F11, BD Biosciences, 557659 7215837<br>2) CD11b, BV510, M1/70, BioLegend, 101245, B360991<br>3) Ly6C, AF700, AL-21, BD Biosciences, 561237, 0293151<br>4) Ly6G, Pacific Blue, 1A8, BioLegend, 127612, B288476<br>5) IA/IE, PerCP/Cy5.5, M5/114.15.2, BD Biosciences, 562363, B253463<br>6) CD80, APC, 16-10A1, BioLegend, 104714, B331465<br>7) F480, BV605, BM8, BioLegend, 123133, 4329685<br>8) CD11c, FITC, HL3, BD Biosciences, 553801, 9352193<br>9) CD45, PE, 30-F11, BD Biosciences, 553801, 2300873<br>10) CD3, PerCP/Cy5.5, 17A2, BD Biosciences, 560591, B207079<br>11) CD11c, PE/Cy7, HL3, BD Biosciences, 561022, 7319586<br>12) Ly6G, BV395, 1A8, BD Biosciences, 563978, 9343809<br>13) CCR7, BV421, 4B12, BD Biosciences, 562675, 1284362<br>14) cKit, PE/Cy7, 2B8, BioLegend, 105814, B205421<br>15) CD16/CD32 FcγIII/II, unconjugated, 2.4G2, BD Biosciences, 553141, 8130843
Validation	For all flow/FACs experiments, positive signal for all antibodies was validated by referencing an unlabeled sample to a single-stain and a fluorescence minus one control. Each antibody was validated by the manufacturer, as indicated on their website, in relevant primary cells or in an antigen-expressing cell lines by comparing positive signal from labeled cells to cells incubated with an isotype control antibody. Antibodies 1-4, 7-11 and 15 have been previously validated and used in publications by our group (Winkler et al. JI, 2020, Winkler et al. JI 2018) and all the antibodies used in this study were validated in initial studies have been cited by multiple papers as demonstrated on the manufacturer's website.

Eukaryotic cell lines

Policy information about cell lines and Sex and Gender in Research

Cell line source(s)	Vero, CCL-81, ATCC
Authentication	Direct source purchase from ATCC.
Mycoplasma contamination	No additional testing for mycoplasma was performed.
Commonly misidentified lines (See ICLAC register)	None

Animals and other research organisms

Policy information about studies involving animals; ARRIVE guidelines recommended for reporting animal research, and Sex and Gender in Research

Laboratory animals	Mouse. Strains: C57BL/6J (B6), Ccr2-/- RFP (B6.129(Cg)-Ccr2tm2.1lfc/J), Ccr7-/- (B6.129P2(C)-Ccr7tm1Rfor/J), in-house generated Ccr2-/- RFP/Ccr7-/- double knockout and Ccr2-/- RFP x Ccr7-/- heterozygous mice.
Wild animals	The study did not involve wild animals.

Reporting on sex	For all flow cytometry, phagocytosis, qRT RNA-seq and survival analyses, experimental groups were mixed-sex and as equally distributed as possible based on the number of male and female mice in each litter. No sex-related phenotype was observed, similar to prior LACV studies.<br>For the RT2 Profiler PCR Array, only male mice were used to minimize sex-specific transcriptional hits as recommended by the manufacturer.
Field-collected samples	Study did not involve samples collected from the field.
Ethics oversight	Mouse experiments were approved by the Rocky Mountain Laboratories (RML) Animal Care and Use Committee and adhered to the National Institutes of Health guidelines and ethical policies. The RML facility is fully accredited by AAALAC International.

Note that full information on the approval of the study protocol must also be provided in the manuscript.

Flow Cytometry

Plots

Confirm that:

- [x] The axis labels state the marker and fluorochrome used (e.g. CD4-FITC).
- [x] The axis scales are clearly visible. Include numbers along axes only for bottom left plot of group (a 'group' is an analysis of identical markers).
- [ ] All plots are contour plots with outliers or pseudocolor plots.
- [x] A numerical value for number of cells or percentage (with statistics) is provided.

Methodology

Sample preparation	At indicated time points for FACS and flow experiments, brain and whole bone marrow was collected into ice cold PBS and for flow cytometry experiments, blood was collected in a 1 mL syringe attached to a 27-gauge 1/2" needle with the needle barrel filled with 1000U/mL heparin. Using a Dounce homogenizer, brains were dissociated and passed through a 100um filter to generate a single cell suspension. Cells were pelleted at 500g and resuspended in 70% Percoll in PBS then underlayed on a 0-30% Percoll step gradient and centrifuged at 500g for 20min. CNS immune cells were recovered at the 30-70% interface, rinsed with PBS and placed on ice to await antibody labeling and fixation. 500ul of whole blood was mixed with 500ul of 2% dextran T500 in PBS and incubated at 37C for 30min to precipitate red bloods cells. The clear upper fraction containing white blood cells was collected and placed on ice. On ice, bone marrow was passed through a 70um filter to generate a single-cell suspension and was then incubated with LIVE/DEAD fixable blue (ThermoFisher) at 1ul of working solution per 10^6 cells for 30min to identify live cells prior to further processing. Whole bone marrow and brain and blood immune cells were incubated with CD16/CD32 FcγIII/II (1:500 brain and 1:200 blood, BD Biosciences, clone 2.4G2) for 30min to block Fc-receptors and subsequently surface immunolabeled with the indicated antibodies (Table 2) at a dilution of 1:200 for 30-45min on ice. Cells were then washed with 3x with PBS, fixed with 2% paraformaldehyde for 30min and washed again with PBS. RFP fluorescence expression in the place of CCR2 on iMOs was observed in Ccr2-/- RFP, DKO and HET mice.
Instrument	Cells were sorted by a FACSAria II (BD Biosciences) or a MACSQuant Tyto (Miltenyi Biotec) and flow cytometry analysis was performed using a BD LSRII or FACsSymphony A5 (BD Biosciences).
Software	BD FACSDiva Software (version 9) was using to collect data and FCS Express Research Edition (version 5) was used for analysis.
Cell population abundance	iMOs accounted for between 35-45% of the infiltrating immune cells into the brains of LACV infected mice as previously reported (Winkler et al. JI, 2018) and between 6-12% of infiltrating immune cells in the brain of HSV infected mice. Purity was established through antibody-specific gating (see next section), iMO-specific transcript expression by sorted cell from LACV and HSV infected mice (Figure 5a and b) and confirmation of consistent expression of CCR2, which is a defining feature of iMOs, in the associated RT2 Profiler PCR array analysis (Supplementary Information, Table 1.)
Gating strategy	FACS gating on brain entailed 1) excluding doublets or cell debris by gating on the linear aspect of the FSC-H/FSC-A, 2) gating on the CD45+ immune cell population, 3) excluding the CD3+ lymphocyte, CD11c+ antigen-presenting cell, CD45/CD11b intermediate microglia and Ly6G+ granulocyte populations, and 4) gating on the Ly6C+ iMO population to collect.<br>Flow gating on brain entailed 1) excluding doublets or cell debris by gating on the linear aspect of the FSC-H/FSC-A, 2) exclusion of CD11c+ and IA/IE+ antigen-presenting cell populations, , 3) gating on the CD45+/CD11b+ population, excluding the D45/CD11b intermediate microglia population 4) excluding the Ly6G+ granulocyte populations and gating on Ly6C+ cells, 5) confirmation that these cells expression intermediate amounts of CD80 and F4/80 or RFP signal if cells from Ccr2-/- RFP, HET or DKO mice. Flow on blood entailed all the same steps except the microglial population was absent. These steps are highlighted in blood samples in Supplementary Figure 1.

- [x] Tick this box to confirm that a figure exemplifying the gating strategy is provided in the Supplementary Information.
